# Research and Remedies

**Published:** 1913-11-01

**Authors:** 


### RESEARCH AND REMEDIES.

### Fatal Eczema in Childhood.

Some very unusual cases of eczema in children are discussed by Dr. P. S. Hichens in a paper read at the provincial meeting of the Section for the Study of Diseases of Children of the Royal Society of Medicine. Out of twenty-eight cases of this disease (in children) admitted to hospital during the last twelve years, no fewer than six have terminated fatally. It has to be borne in mind, of course, that only very severe cases of eczema would be likely to be admitted at all; but even allowing for that, the proportion of fatalities is astonishing. Curiously enough, in two of the cases the skin eruption was yielding steadily to treatment, and everything seemed to be going on well, when a sudden collapse set in which proved rapidly fatal. Discussing the cases, Dr. Hichens thinks that the suspicion of the deaths being due to metallic poisoning by absorption of zinc or lead from the lotions and ointments used can be set aside as untenable. He confesses himself baffled in the attempt to explain these deaths, especially the two referred to above; and is unwilling to invoke the *status lymphaticus*, of which there was no evidence. In this connection it is interesting to recall the superstition current among the working classes that a skin rash should not be too quickly cured, lest the "poison be driven inwards" with disastrous results. Possibly there is a grain of truth in this unorthodox view.

### The Use of Pituitary Extract.

The use of pituitary extract has become so frequent that there is some danger of the limitations of this powerful (and for many conditions admirable) drug being overlooked. First and foremost stands the oxytocic action of pituitary extract, which is perhaps the most valuable of its properties. Beyond question, an intramuscular injection of pituitary extract causes within a few minutes powerful uterine contractions, and this action has proved of immense value in obstetric practice in the treatment of post-partum hæmorrhage. As in the case of ergot, the result of administering the drug before delivery may not be so good, for if pelvic contraction or other physical obstacle be present, the result of violently increasing the uterine contractions may be evil instead of good. Thus, in the *Zentralblatt für Gynäk.*, Herz reports a case of rupture of the uterus caused in this way; fortunately the patient recovered. He dwells upon the fact that pituitrin should not be used until full dilatation of the cervix has occurred. Other complications, besides rupture of the uterus, that have been reported when this rule is neglected include post-partum atony of the uterus, asphyxia neonatorum, collapse with tinnitus and vertigo, eclampsia, tetanic uterine contraction, contraction of the previously dilated os, and premature separation of the placenta. The mention of collapse reminds us of the fact that pituitary extract has been a good deal given for shock, though the reports are by no means as unanimous in favour of it as in the case of post-partum hæmorrhage. The present writer has seen two cases in which pituitary extract thus given has seemed to aggravate existing symptoms for a few minutes, after which rapid improvement set in; and this experience he has heard corroborated by nurses. The lesson is caution in the use of a powerful drug whose exact effects and limitations are not yet ascertained.

